# Typification of Zapałowicz’s names in Aconitum section Aconitum

**DOI:** 10.3897/phytokeys.58.7110

**Published:** 2016-01-12

**Authors:** Klaudia Wacławska-Ćwiertnia, Józef Mitka

**Affiliations:** 1 Polish Academy of Science, Władysław Szafer Institute of Botany, 31-512 Kraków, Lubicz 46, Poland; 2 Jagiellonian University, Institute of Botany, Botanical Garden, 31-501 Kraków, Kopernika 27, Poland

**Keywords:** Aconitum berdaui, Aconitum bucovinense, Carpathians, nomenclature, taxonomy, typification

## Abstract

Hugo Zapałowicz described and named 27 taxa in Aconitum sect. Aconitum. Their names are typified here. Two of them (*Aconitum berdaui*, *Aconitum bucovinense*) are deemed correct for currently accepted species of the Carpathians, 24 are reduced to synonymy under five taxa, and for one no original material has been located. The correct place and exact date of their publication, which differs from those usually assumed, have been ascertained by bibliographic verification and the study of archival documents.

## Introduction

The species concepts and classification in Aconitum L. sect. Aconitum, as represented in the Carpathians, were proposed and discussed by Mitka (2000, 2002, 2003), Starmühler and Mitka (2001) and Novikoff and Mitka (2011). Cytogenetic information has been added by Ilnicki and Mitka (2009). Here, we consider the type specimens of relevant names published by Hugo Zapałowicz (1852–1917), applying the rules of the Melbourne Code (McNeill et al. 2011) and based on herbarium material deposited in KRAM.

To date it has been a generally accepted practice to cite all nomenclatural novelties that appear in Zapałowicz’s “Conspectus florae Galiciae” from that Conspectus. However, we will show that, as correctly stated by Stafleu and Cowan (1988: 521) then confirmed by Köhler (2002), they should instead be cited from corresponding articles in “Rozprawy Wydziału Matematyczno-Przyrodniczego Akademii Umiejętności. Seria III. Dział B. Nauki biologiczne” (hereafter “Rozprawy”), which were published several months earlier than the “Conspectus”. We verified that information by checking the available evidence (including printers’ bills) related to the publication of the “Conspectus” and “Rozprawy” issues. We found that the date of publication of the relevant portion of the “Conspectus” is 22 December 1908, whereas for the “Rozprawy” issue it is 5 May 1908. Effective publication of the *Aconitum* names in question took place on the earlier date.

## Materials and methods

Effective and valid publication of the *Aconitum* names that appear in the first fascicle of the “Rozprawy” (Zapałowicz 1908a) and in the second volume of the “Conspectus” (Zapałowicz 1908b) were assessed by study of the protologues. The corresponding herbarium material deposited in KRAM (Thiers 2011, http://sweetgum.nybg.org/science/), where a full set of Zapałowicz’s plant collections is deposited, has been studied. In typifying these names we followed the rules of the International Code of Nomenclature for algae, fungi and plants (McNeill et al. 2012) and the recommendation made by McNeill (2014). In cases where a single specimen is known that was used by Zapałowicz, that particular specimen is nevertheless considered a lectotype, with the additional qualification: “or perhaps holotype”.

Accepted names of the taxon, determined in conformity with Mitka (2003), appear in ***bold italics***. If different from the name here typified, they are added at the end of the entry [in square brackets, when they are heterotypic].

## Results

In his treatment of Aconitum sect. Aconitum for the “Conspectus”, Zapałowicz described and named 27 taxa: two hybrids, 9 varieties and 16 formae. Their names, with one exception, are typified here. Two hybrids (*Aconitum berdaui*, *Aconitum bucovinense*) are deemed correct for currently accepted species of the Carpathians, 24 are reduced to synonymy under five taxa, and for one no original material has been located.

***Aconitum berdaui*** Zapał. in Rozpr. Wydz. Mat.-Przyr. Akad. Umiej., Dział B. Nauki Biol. 48: 88. 1908, *pro hybr*. (http://ipni.org/urn:lsid:ipni.org:names:707195-1:1.2.2.1.1.1). Protologue text: “In regione subalpina Tatrorum: in valle Kościeliska et altero loco non indicato (*Berdau*), Podspady ad Jaworzynka (*Rogalski*)”. Lectotype (designated here): “Dol. Kościeliska, ?.08.1855, leg. *Berdau*, det. *Zapałowicz* (16.02.1908), KRAM! (133482). Other syntypes: (1) “Podspady nad Jaworzynką, 25.07. (1878?), *A. Rogalski*” – KRAM! (133481); (2) “sine die, leg. *Berdau*, det. *Zapałowicz* (16.01.1908)” – KRAM! (133483).

***Aconitum bucovinense*** Zapał. in Rozpr. Wydz. Mat.-Przyr. Akad. Umiej., Dział B. Nauki Biol. 48: 89-90. 1908, *pro hybr*. (http://ipni.org/urn:lsid:ipni.org:names:707221-1:1.3). Protologue text: „In montanis et subalpinis Bucovinae australis: Jakobeny, Pojana niegri prope Dorna Kandreny (*Rehman*)”. Lectotype (designated here, or perhaps holotype): “Dr *A. Rehman*: Exsiccata Florae Galiciensis; *Aconitum*; Jakobeni na Bukowinie [no date]; Nr LXXVI/3532” – KRAM! (132396).

Aconitum napellus f. abnorme Zapał. in Rozpr. Wydz. Mat.-Przyr. Akad. Umiej., Dział B. Nauki Biol. 48: 84. 1908. Protologue text: „Miętusia w Tatrach (*Kulczyński*)”. Lectotype (designated here, or perhaps holotype): „Miętusia, 26.07.1875 r., leg. *W. Kulczyński*” – KRAM! (132273), first synonimized by Mitka (2003). – [***Aconitum firmum*** Rchb. subsp. ***firmum***].

Aconitum napellus f. amoenum Zapał. in Rozpr. Wydz. Mat.-Przyr. Akad. Umiej., Dział B. Nauki Biol. 48: 87. 1908. Protologue text: „Alpy Rodneńskie: Piatra rei u stóp skał wapiennych 1300-1350 m (*Zapałowicz*)”. Lectotype (designated here, or perhaps holotype): „Piatra rei. Alpy Rodneńskie, 15.08.1907, leg. et det. *H. Zapałowicz*” – KRAM! (132300). – [***Aconitum czarnohorense*** (Zapał.) Mitka].

Aconitum napellus var. babiogorense Zapał. in Rozpr. Wydz. Mat.-Przyr. Akad. Umiej., Dział B. Nauki Biol. 48: 85. 1908. Protologue text: „W krainie kosodrzewu Babiej Góry w Kościółkach 1565 m i t. d., wyjątkowo nad potokami nieraz bardzo nizko, np. nad Markowym Potokiem 725 m (*Zapałowicz*); a także na szczycie 1725 m (*Bobek*); Kuźnice w Zakopanem (*Jabłoński*). Okaz z Czarnej Hory (*Rehman*) bez bliższego oznaczenia miejscowości, planta cum filamentis glaberrima, bez żadnej wątpliwości tu należy. Ze względu na daleki wschód jest okaz ten bardzo ważny i pouczający; zacieśnia on związek między zachodnio – karpacką odmianą babiogorense m. a wschodnio-karpacką odm. swidovense sekcyi II tak samo, jak niektóre formy pośrednie wymienione pod var. czarnohorense łączą tę ostatnią odmianę z zachodnio-karpacką odmianą tatrense m. sekcyi III”. Lectotype (designated here): „Babia Góra nad Markowym Potokiem, leg. et det. *H. Zapałowicz* (2.08.1877)” – KRAM! (132263). Other syntypes: (1) „Dyablak, ?.08.1876, leg. *K. Bobek*” – KRAM! (132259); (2) „Zakopane, Kuźnice, 17.07. ?, leg. *W. Jabłoński*”, KRAM! (132290); (3) „Czarna Hora, sine die” – KRAM! (132267). – [***Aconitum firmum*** Rchb. subsp. ***firmum***].

Aconitum napellus var. bidgostianum Zapał. in Rozpr. Wydz. Mat.-Przyr. Akad. Umiej., Dział B. Nauki Biol. 48: 83. 1908. Protologue text: „Koło Bydgoszczy”. Lectotype (designated here, or perhaps holotype): “Okolice Bydgoszczy, Czyszkówko, sine die, leg. *Trabandt*, det. *Zapałowicz* (12.02.1908)” – KRAM! (132292). – [***Aconitum bucovinense*** Zapał.].

Aconitum napellus var. carpaticum Zapał. in Rozpr. Wydz. Mat.-Przyr. Akad. Umiej., Dział B. Nauki Biol. 48: 84. 1908. Protologue text: „Babia Góra z niższej dziedziny lasów (*Bobek*), z innego miejsca (*Kulczyński*). Mała Łąka w Tatrach (*Kulczyński*)”. Lectotype (designated here): „Mała Łąka, 13.08.1875, leg. et det. *W. Kulczyński*” – KRAM! (132272). Other syntype: „Babia Góra, ?.08.1876, leg. *W. Kulczyński*” – KRAM! (132270). – [***Aconitum firmum*** Rchb. subsp. ***firmum***].

Aconitum napellus var. czarnohorense Zapał. in Rozpr. Wydz. Mat.-Przyr. Akad. Umiej., Dział B. Nauki Biol. 48: 86. 1908.(http://ipni.org/urn:lsid:ipni.org:names:77062909-1:1.2). Protologue text: „W Górach Pokucko-Marmaroskich, szczególnie na Czarnej Horze i w Alpach Rodneńskich w krainie kosodrzewu często, najwyżej tam 2010-2030 m; liczne okazy z Czarnej Hory i z Pietrosu w Alpach Rodneńskich (*Zapałowicz*)”. Lectotype (designated here): „Góry Pokucko-Marmaroskie, Pietrosz, 8.08.1880, leg. et det. *H. Zapałowicz*” - KRAM! (246853). Other syntypes: (1) „Pietrosz Kr. Kos., 25.07.1905, *H. Zapałowicz*” – KRAM! (132297); (2) „Góry Pokucko-Marmaroskie, leg. et det. *H. Zapałowicz*, 28.07.1880” – KRAM! (246854); (3) „Czarna Hora, 18.09.1881, leg. et det. *H. Zapałowicz* (12.02.1908)” – KRAM! (132277). – (≡ ***Aconitum czarnohorense*** (Zapał.) Mitka).

Aconitum napellus f. glabratum Zapał. in Rozpr. Wydz. Mat.-Przyr. Akad. Umiej., Dział B. Nauki Biol. 48: 87. 1908. Protologue text: „Ihrowiszcze (*Zipser*), Howerla (*Witwicki*), Pietrosu na Piatra alba (*Zapałowicz*), Dorna Watra na Bukowinie (*Rehman*). Lectotype (designated here): Góry Pokucko-Marmaroskie, Pietrosz, 29.08.1880, leg. et det. *H. Zapałowicz*” – KRAM! (246852). Other syntypes: (1) „Ihrowiszcze granica Węgier, ?.?.1865, leg. *S. Zipser*” – KRAM! (132255); (2) „Howerla koło Żabiego, ?.?.1865, leg. *Witwicki*” – KRAM! (132254); (3) „Dorna Watra na Bukowinie”, sine die, *Rehman*” – KRAM! (132266). – [***Aconitum czarnohorense*** (Zapał.) Mitka].

Aconitum napellus f. grofense Zapał. in Rozpr. Wydz. Mat.-Przyr. Akad. Umiej., Dział B. Nauki Biol. 48: 84. 1908. – Protologue text: „W Karpatach nad Łomnicą: p. Kotełec pod Grotą (*Wołoszczak*)”. Lectotype (designated here, or perhaps holotype): „W Karpatach nad Łomnicą: p. Kotełec pod Grotą, 20.07.1889, leg. *E. Wołoszczak*, det. *H. Zapałowicz*” – KRAM! (132282). – [***Aconitum firmum*** Rchb. subsp. ***firmum***].

Aconitum napellus f. howerlanum Zapał. in Rozpr. Wydz. Mat.-Przyr. Akad. Umiej., Dział B. Nauki Biol. 48: 87. 1908. Protologue text: „Howerla i z innego szczytu w Czarnej Horze (*Wołoszczak*)”. Lectotype (designated here): „Czarna Hora na Negrowin, 2.08.1888, leg. *E. Wołoszczak*” – KRAM! (132276a). Other syntype: „Howerla, 26.07.1886, leg. *E. Wołoszczak*, *em. H. Zapałowicz*” – KRAM! (132269). – [***Aconitum czarnohorense*** (Zapał.) Mitka].

Aconitum napellus f. latisectum Zapał. in Rozpr. Wydz. Mat.-Przyr. Akad. Umiej., Dział B. Nauki Biol. 48: 84. 1908. – Protologue text: „Mała Łąka w Tatrach (*Kulczyński*)”. Lectotype (designated here, or perhaps holotype): „Mała Łąka, 13.08.1875, leg. *W. Kulczyński*” – KRAM! (132257), first synonimized by Mitka 2003. – [***Aconitum firmum*** Rchb. subsp. ***firmum***].

Aconitum napellus var. lomnicense Zapał., Rozpr. Wydz. Mat.-Przyr. Akad. Umiej., Dział B. Nauki Biol. 48: 87-88. 1908. Protologue text: „W Karpatach Wschodnich: nad Łomnicą “przy stawie Grofy” (*Wołoszczak*), więc już dość nizko, mniej więcej w dziedzinie górskiej”. Lectotype (designated here, or perhaps holotype): „Przy stawie Grofy, 31.08.1890, leg. *E. Wołoszczak*, det. *H. Zapałowicz* (16.02.1908)” – KRAM! (132286). – [***Aconitum nanum*** (Baumg.) Simonk.].

Aconitum napellus f. minimum Zapał. in Rozpr. Wydz. Mat.-Przyr. Akad. Umiej., Dział B. Nauki Biol. 48: 87. 1908. Protologue text: „Na Czarnej Horze: Pop Iwan koło 2000 m (*Zapałowicz*)”. Type not designated (no material traced). – Identity uncertain.

Aconitum napellus f. puberulum Zapał. in Rozpr. Wydz. Mat.-Przyr. Akad. Umiej., Dział B. Nauki Biol. 48: 84-85. 1908. Protologue text: „Babia Góra w lasach pod Djablakiem koło 1160 m (*Zapałowicz*)”. Lectotype (designated here, or perhaps holotype) „1160 m, 24.07.1906, *H. Zapałowicz*” – KRAM! (132299), first synonimized by Mitka 2003. – [***Aconitum firmum*** subsp. ***moravicum*** Skalický].

Aconitum napellus f. rodnense Zapał. in Rozpr. Wydz. Mat.-Przyr. Akad. Umiej., Dział B. Nauki Biol. 48: 87. 1908. Protologue text: „W krainie kosodrzewu Alp Rodneńskich, widocznie przeważnie lub wyłącznie na wapieniach: Piatra rei 1300-1400 m, Galacz 1760-1850 m (*Zapałowicz*)”. Lectotype (selected here): „Alpy Rodneńskie, Galacz, 15.08.1907, leg. et det. *H. Zapałowicz*” – KRAM! (132301). Other syntype: „Piatra Alpy Rodneńskie, 15.08.1907, leg. et det. *H. Zapałowicz*” – KRAM! (132302). – [***Aconitum czarnohorense*** (Zapał.) Mitka].

Aconitum napellus var. silesiacum Zapał. in Rozpr. Wydz. Mat.-Przyr. Akad. Umiej., Dział B. Nauki Biol. 48: 84. 1908. Protologue text: „Barania w Karpatach u źródeł Wisły na Śląsku (*Wołoszczak*)”. Lectotype (designated here, or perhaps holotype): „Na Baraniej Górze w Beskidzie, okoł. 1000 m, Galicya, 10.07.1895, leg. *E. Wołoszczak*, 13.07.1908, rev. *H. Zapałowicz*” – KRAM! (132291), first synonimized by Mitka 2003. – [***Aconitum firmum*** subsp. ***moravicum*** Skalický].

Aconitum napellus f. subincisum Zapał. in Rozpr. Wydz. Mat.-Przyr. Akad. Umiej., Dział B. Nauki Biol. 48: 86-87. 1908. – Protologue text: „Ukiernia w Karpatach na górnem porzeczu Łomnicy (*Wołoszczak*)”. Lectotype (designated here, or perhaps holotype): „Na północno-zachodnim szczycie Ukierni przy Łomnicy, Karpaty, 01.08.1890, *E. Wołoszczak*” – KRAM! (132285). – [***Aconitum czarnohorense*** (Zapał.) Mitka].

Aconitum napellus f. turkulense Zapał. in Rozpr. Wydz. Mat.-Przyr. Akad. Umiej., Dział B. Nauki Biol. 48: 86. 1908. Protologue text: „W krainie kosodrzewu Czarnej Hory pod Turkułem (*Zapałowicz*)”. Lectotype (designated here, or perhaps holotype): „Czarna Hora, 21.08.1880. *H. Zapałowicz*, det. *H. Zapałowicz* (12.02.1908)” – KRAM! (132278), first synonimized by Mitka (2003). – [***Aconitum czarnohorense*** (Zapał.) Mitka].

Aconitum napellus f. subfissum Zapał. in Rozpr. Wydz. Mat.-Przyr. Akad. Umiej., Dział B. Nauki Biol. 48: 85. 1908. – Protologue text: „Kościeliska w Tatrach (*Krupa*)”. Lectotype (designated here, or perhaps holotype): „Kościelisko, Tatry, sine die, *Krupa*” – KRAM! (132284). – [***Aconitum firmum*** Rchb. subsp. ***firmum***; first synonymized by Mitka (2003)].

Aconitum napellus var. subtatrense Zapał. in Rozpr. Wydz. Mat.-Przyr. Akad. Umiej., Dział B. Nauki Biol. 48: 83-84. 1908. Protologue text: Tatry, w strefie widocznie niższej, podalpejskiej, okazy zebrane przez *Berdaua*, *Rehmana* i *Jabłońskiego*, bez bliższego oznaczenia miejscowości. Lectotype (designated here): „Widoczne z Tatr, *Berdau*” – KRAM! (132287). Other syntypes: (1) „Widoczne z Tatr, sine die, leg. *A. Rehman*, det. *H. Zapałowicz* (12.02.1908)” – KRAM! (132268); (2) „Czarna Hora, najprędzej Tatry, *H. Zapałowicz*” – KRAM! (132265); (3) Tatry wszędzie prawie, ?.07.1862, *W. Jabłoński*” – KRAM! (132289). – [***Aconitum firmum*** Rchb. subsp. ***firmum***].

Aconitum napellus f. subvestitum Zapał. in Rozpr. Wydz. Mat.-Przyr. Akad. Umiej., Dział B. Nauki Biol. 48: 85. 1908. Protologue text: „Szpyci w Czarnej Horze (*Śleńdziński*)”. Lectotype (designated here, or perhaps holotype): „Czarna Hora, Szpyci, 21.08.1875, leg. *Śleńdziński*” – KRAM! (132260). – [***Aconitum czarnohorense*** (Zapał.) Mitka].

Aconitum napellus var. swidovense Zapał. in Rozpr. Wydz. Mat.-Przyr. Akad. Umiej., Dział B. Nauki Biol. 48: 85. 1908. Protologue text: „Na północny-zachód od Czarnej Hory w Górach Swidowskich: Dragobrat. Bliźnica 1700 m i t. d. (*Zapałowicz*)”. Lectotype (designated here, or perhaps holotype): „G. Świdowskie, Dragobrat, Bliźnica, 10.08.1882, leg. et det. *H. Zapałowicz*” – KRAM! (132385). – [***Aconitum czarnohorense*** (Zapał.) Mitka].

Aconitum napellus var. tatrense Zapał. in Rozpr. Wydz. Mat.-Przyr. Akad. Umiej., Dział B. Nauki Biol. 48: 86. 1908. Protologue text: „Okazy ze Świnicy (*Kotula*) i z innego miejsca w Tatrach (*Janota*). Według *Kotuli* (l.c.) w krainie kosodrzewu Tatr w ogóle bardzo często, sięga po 2180-2210 m”. Lectotype (designated here): „Tatry (?), ?.?.1877, leg. Dr. *E. Janota*, det. *Zapałowicz*” – KRAM! (132261). Other syntype: „Tatry pod szczytem Świnicy, 30.07.1880, det. H. *Zapałowicz* (12.02.1908)” – KRAM! (132279). – [***Aconitum firmum*** Rchb. subsp. ***firmum***].

Aconitum napellus f. tenuisectum Zapał. in Rozpr. Wydz. Mat.-Przyr. Akad. Umiej., Dział B. Nauki Biol. 48: 86. 1908. Protologue text: „Dział między Howerlą a Pietroszem, blizko drugiego na skałkach jurajskich koło 1550 m (*Zapałowicz*)”. Lectotype (designated here, or perhaps holotype): „Czarna Hora, Pietrosz, 5.08.1906, leg. et. det. *H. Zapałowicz*” – KRAM! (132298), first synonimized by Mitka 2003. – [***Aconitum czarnohorense*** (Zapał.) Mitka].

Aconitum napellus f. vestitum Zapał. in Rozpr. Wydz. Mat.-Przyr. Akad. Umiej., Dział B. Nauki Biol. 48: 85. 1908. – Protologue text: „Pilsko, widocznie z krainy kosodrzewu (*Krupa*), 2 okazy”. Lectotype (designated here, or perhaps holotype): “Pilsko, ?.08.1878, leg. *Krupa*” – KRAM! (132283), first synonimized by Mitka 2003. – [***Aconitum firmum*** subsp. ***moravicum*** Skalický].

Aconitum napellus f. zeleminum Zapał. in Rozpr. Wydz. Mat.-Przyr. Akad. Umiej., Dział B. Nauki Biol. 48: 87. 1908. Protologue text: „Karpaty na południe od Skolego: Zełemin, Wysoka-Ihrowiszcze (*Wołoszczak*)”. Lectotype (designated here): „Pod szczytem Wysokiej (Ihrowiszcze) w okolicy Łomnicy, Karpaty, 27.07.1889, leg. *E. Wołoszczak*, det. *H. Zapałowicz*” – KRAM! (132280). Other syntype: „Zełemin, ?.08.1891, *E. Wołoszczak*” – KRAM! (132288). – [***Aconitum czarnohorense*** (Zapał.) Mitka].

## Discussion

In 1907 Zapałowicz started to lay down his *opus magnum*, “Conspectus florae Galiciae”. As detailed by Stafleu and Cowan (1988: 521), this work was first published in fascicles in the “Rozprawy” (e.g., Zapałowicz 1908a), under its alternative Polish title “Krytyczny Przegląd Roślinności Galicji” (“Przegląd”). In total, from 1907 to 1914 thirty fascicles were published. Fascicles 1–22 were subsequently reprinted and published in book form in three volumes. Volume 1 consisting of fascicles 1–7, was published in 1906; volume 2 (Zapałowicz 1908b), comprising fascicles 8–14, in 1908; and volume 3, with fascicles 15–22, in 1912. Fascicles 23–30, which were published in 1912–1914, were not reprinted in book form.

In order to establish the effective date of publication of the “Przegląd” and volume 2 of “Conspectus florae Galiciae” in which the treatment of *Aconitum* is published, the first author checked the available documents in the archives of the library of the Institute of Botany of the Polish Academy of Sciences (*Polska Akademia Nauk*, PAN) (1), the Academic Library of the Academy of Sciences and Letters (*Polska Akademia Umiejętności* PAU) (2), the Archives of the Science of the PAN and PAU (3), the Jagiellonian University Archives (4) and the Jagiellonian Library (5).

Each fascicle of the “Rozprawy” was published subsequent to a meeting of the Division of Mathematics and Natural Sciences of PAU. At the end of each year, the fascicles of were put together and issued as an annual volume (“Przegląd”) with its own title page and table of contents. Unfortunately wrappers mostly removed front pages and tables of content from individual fascicles. In the Jagiellonian Library in Cracow (ID number 28061 III) we found a single vol. 48 of the “Rozprawy” fasicles with the original front and back wrapper sheets of the fascicles have been preserved. Three “Rozprawy” fascicles were published in 1908. The first, with seven articles, includes parts XII and XIII of the “Przegląd”; the second contains nine articles and the third and last, six. Part XII of the “Przegląd” was presented to the Division of Mathematics and Natural Sciences at its meeting on 2 March 1908; the minutes of the meeting on 4 May 1908 state that part XIII was approved and sent to the printers. On the following day (5 May) the Jagiellonian University Printing House billed PAU for the first fascicle of the “Rozprawy”, in which both parts are included. The bill for the second fascicle is dated 7 August 1908; that for the third fascicle, 22 December 1908. The word „wydrukowano” [printed] appears on all bills, so that the date of effective publication of parts XII and XIII of “Przegląd” may be accepted to be 5 May 1908. The Jagiellonian Printing House billed PAU for volume 2 of the “Conspectus” on 22 December 1908. Effective publication of the *Aconitum* names in question took place on the earlier date.
